# Exploration of Sorafenib Influences on Gene Expression of Hepatocellular Carcinoma

**DOI:** 10.3389/fgene.2020.577000

**Published:** 2020-10-08

**Authors:** Huancheng Wang, Jia Liu

**Affiliations:** First Teaching Hospital of Tianjin University of Traditional Chinese Medicine, Tianjin, China

**Keywords:** hepatocellular carcinoma, sorafenib, GO and KEGG functional enrichment, protein–protein interaction analysis, hepatocellar carcinoma

## Abstract

**Purpose:**

The aim of this study was to develop a comprehensive differential gene profile for hepatocellular carcinoma (HCC) patients treated with sorafenib.

**Methods:**

The RNA sequencing data and miRNA sequencing data of 114 HCC patients treated with sorafenib only and 326 HCC control patients treated without any chemotherapeutic drugs were studied using differential expression, functional enrichment, and protein–protein interaction analysis.

**Results:**

Compared with HCC patients without any chemotherapy drugs, the sorafenib-treated patients develop 66 differentially expressed genes (DEGs), including 12 upregulated genes and 54 downregulated genes. Additionally, three differentially expressed miRNAs (DEMs) also show specific expression pattern. With further analysis, five primary genes including HTR2C, TRH, AGTR2, MCHR2, and SLC6A2 as well as three miRNAs (hsa-miR-4445, hsa-miR-466, and hsa-miR-2114) have been suggested as the potential targets for sorafenib. The specific gene expression of five genes has been validated in clinical HCC patients by ELISA method. Gene Ontology (GO) and Kyoto Encyclopedia of Genes and Genomes (KEGG) functional enrichment analyses indicate several significantly enriched biological processes (BPs), cellular components (CCs), and molecular functions (MFs).

**Conclusion:**

Since sorafenib is becoming increasingly important in HCC treatment clinically, this study will help us understand the potential targets and eliminate diverse existing side effects of it as well as explore several potential clinical biomarkers with comprehensive analysis of differential gene expression profile.

## Introduction

Hepatocellular carcinoma (HCC) is the most common primary malignancy of the liver, comprising 75–85% of cases of liver cancer (El-Serag, 2011). At the same time, it is also the second leading cause of cancer-related deaths worldwide, accounting for nearly 841,000 new cases and 782,000 deaths annually (Lurje et al., 2019). The hepatitis B virus (HBV) and hepatitis C virus (HCV) are critical causes of viral hepatitis that lead to the development of HCC (Colquhoun, 2016). The pathogenesis of virus-induced HCC is suggested to involve several mechanisms, such as HBV-DNA integration into host genetic machinery, DNA methylation, and oxidative stress (Li et al., 2015; Forner et al., 2018). The liver chronic illness results from persistence of the virus into the host cells via various receptor-mediated mechanisms that include infection of immune defense control centers, viral inhibition of antigen presentation, selective immune suppression, downregulation of viral gene expression, and viral mutations that functionally incapacitate virus-specific T cells from recognizing the HBV antigen (Grandhi et al., 2016). Even though viral hepatitis from HBV and HCV is highly associated with HCC, there are still some non-viral risk factors that can induce the development of HCC. Diabetes mellitus, alcohol abuse, cardiovascular disease, liver inflammation, obesity, dyslipidemia, and non-alcoholic fatty liver disease (NAFLD) are some other major contributors to HCC development (Schlachterman et al., 2015; Golabi et al., 2019; Kudo, 2019). If detected very early, HCC can be cured with an excellent long-term prognosis, where the principal treatment options would be surgical resection or liver transplantation (Greten et al., 2019). However, for the majority of patients, HCC is detected at a late stage where surgical cure is no longer an option. Most patients will therefore need chemotherapy, which works by destroying cancer cells and inhibiting the proliferation of new cancer cells via the use of chemical agents (Lurje et al., 2019). Sorafenib, also known as Nexavar, the most popularly used chemotherapeutic agent to treat HCC recently, is an oral multikinase inhibitor that efficiently inhibits tumor cell proliferation and angiogenesis and promotes tumor cell apoptosis (Feng et al., 2019; Bertacco et al., 2020). Sorafenib was developed by the Bayer and Onyx companies and was originally approved by the Food and Drug Administration (FDA) for the treatment of advanced renal cell carcinoma in 2006. One year later, it was demonstrated to be a unique target drug for HCC (Hu et al., 2019). It functions as a small multi-tyrosine kinase inhibitor that blocks Raf kinase, vascular endothelial growth factor (VEGF), and platelet-derived growth factor (PDGF) (Nagai et al., 2019). In addition, sorafenib can suppress angiogenesis through targeting of the hepatocyte factor receptor (c-Kit), Fms-like tyrosine kinase (FLT-3), VEGF receptor VEGFR-2, VEGFR-3, PDGF receptor (PDGFR-β), and other tyrosine kinases such as 6 and 7 (Santos et al., 2019; Mammatas et al., 2020; Pearson et al., 2020). It is worth noting that sorafenib is found to be efficient in inhibiting tumor cell growth not only in HCC but also in other tumor cells such as breast cancer MDA-MB-231, pancreatic BxPC3 cells, and colon cancer HCT116 (Labeur et al., 2019). Despite that sorafenib has opened a window of hope for effective agents to treat HCC, the overall outcomes remain inferior. It is suggested to have diverse side effects including diarrhea, weight loss, and hand–foot skin reactions (Pathania, 2019). Additionally, some HCC patients are reported to develop drug resistance to sorafenib (Raoul et al., 2019).

Based on the fact of unknown cause as well as unclear mechanisms underlying sorafenib, its role in the clinic has been greatly limited. To address these issues, in this study, differential expression analysis was performed on HCC patients treated with or without sorafenib. Using a combination of bioinformatics and machine learning method, we comprehensively explore the potential target genes and precise correlated molecular pathways of sorafenib, which provides a potential guidance for the precise administration of sorafenib as well as initiates a tremendous help for HCC patients.

## Materials and Methods

### Study Population

The RNA sequencing data and miRNA sequencing data as well as clinical information from 340 patients with HCC was downloaded from The Cancer Genome Atlas (TCGA)^1^. Among them, 114 HCC patients were treated with sorafenib only, and 326 HCC control patients were treated without any chemotherapeutic drugs.

### Differential Expression Analysis

The edgeR package in R language was performed to analyze differentially expressed genes (DEGs) and differentially expressed miRNAs (DEMs) between diverse groups. The absolute values of logarithmic transformed differential expression multiples (Log2FC) >1 and *P*-value < 0.05 were used as criteria for screening (Robinson et al., 2010).

The *P*-value used represents adjusted *P*-values [false discovery rate (FDR)] considering multiple comparison criteria, which were analyzed against all the genes and miRNA expressions here together.

Meanwhile, the (Log2FC) (used for DEG and DEM screening) stands for an absolute value for different selected targets.

### Functional Enrichment Analysis

The ClusterProfiler package in R language was processed for Gene Ontology (GO) analysis [including biological process (BP), molecular function (MF), and cellular component (CC)] as well as Kyoto Encyclopedia of Genes and Genomes (KEGG) pathway enrichment analysis (Yu et al., 2012). A value of *P* < 0.05 was considered as statistically significant.

### Protein–Protein Interaction Networks

The STRING database is a database that analyzes and predicts the functional protein–protein connections. We use the STRING^2^ (version 11.0) to analyze the functional connections and interactions of candidate proteins (Szklarczyk et al., 2019), of which the interaction pairs with a combined score greater than or equal to 0.4 (confidence score ≥0.4) were retained. The Cytoscape (version 3.7.2) was performed to visualize the protein–protein interaction (PPI) network (Shannon et al., 2003). The network modules reserve specific biological meanings, which are usually the core of protein networks. We process the molecular complex detection method (MCODE) plug-in in Cytoscape software to identify significant clustering modules, using MCODE score >2 as the threshold.

### Prediction of MiRNA Target Genes

The target genes of miRNAs were predicted through the miRDB^4^ (version 6.0) database (Chen and Wang, 2020). Meanwhile, the Cytoscape (see “text footnote 3,” version 3.7.2) was performed to visualize the miRNA–mRNA regulatory network.

### ELISA Analysis of Selected Genes

A total of 183 HCC patients diagnosed by pathology in Tianjin Medical University Cancer Institute and Hospital from January 2018 to December 2019 were randomly selected and collected. In order to exclude the influence of different tumor stages on the results, only 120 patients with stage II were selected for the study. There were 78 males and 42 females. The patients were randomly divided into two groups: the group treated with sorafenib for HCC patients and the group without treatment for HCC patients, with 60 cases in each group. This study is in line with the medical ethics standards and approved by the hospital ethics committee. Patients or their families gave informed consent for all treatments and testing.

The concentrations of potential candidate genes were determined by ELISA double-antibody sandwich method. The specific operation was carried out in strict accordance with the instructions of the kit (Abcam Company, United States). The experimental results were repeated three times independently and were tested by statistical methods.

## Results

### Differentially Expressed mRNA and MiRNA

**FIGURE 1:**
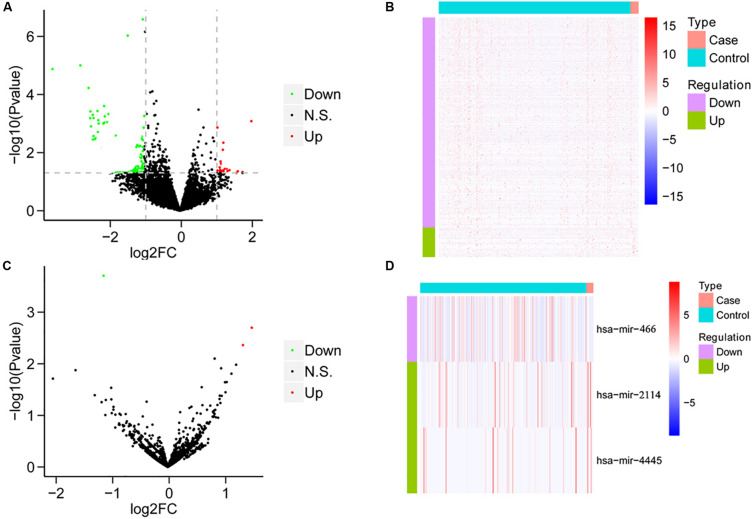
The results of differential analysis of mRNA and miRNA. **(A)** Volcano diagram of differentially expressed mRNA. The horizontal axis is the Log2FC value, and the vertical axis is –log10 (*P*-value). The red color represents upregulation, green represents downregulation, and black represents no significant difference. **(B)** Heat map of differentially expressed mRNA. Behavioral genes are listed as samples. Red represents high expression, and blue represents low expression. **(C)** Volcano map of differentially expressed miRNA. **(D)** The heat map of differentially expressed miRNA expression.

We compared the mRNA expression of HCC patients treated only with sorafenib and HCC patients without any chemotherapy drugs based on the expression levels of all mRNAs. Compared with HCC patients without any chemotherapy drugs, the sorafenib-treated patients demonstrated 66 DEGs, including 12 upregulated genes and 54 downregulated genes (Figures 1A,B). On the other hand, the miRNA expression was analyzed between the two groups. Compared with HCC patients without any chemotherapeutic drugs, patients treated with sorafenib alone displayed three DEMs, including two upregulated miRNAs and one downregulated miRNAs (Figures 1C,D).

### Target Genes of MiRNA

We used the miRDB database to predict target genes of the three DEMs and identified a total of 1,615 target genes. Among the DEM target genes (1,615 target genes), five genes are specifically expressed in HCC patients treated with sorafenib compared with the ones treated without any chemotherapy drugs.

### Gene Ontology and Kyoto Encyclopedia of Genes and Genomes Functional Enrichment Analysis

**FIGURE 2:**
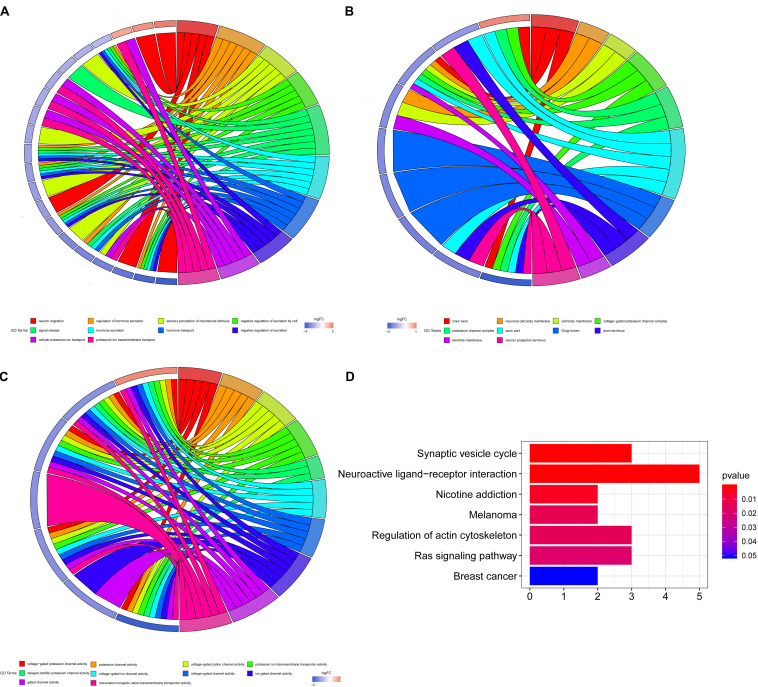
Enrichment analysis of Gene Ontology (GO) and Kyoto Encyclopedia of Genes and Genomes (KEGG). **(A)** The top 10 most enriched biological process (BP) entries. The right half circle is the 10 enriched BP terms, which are represented by different colors; the left half circle is the enriched mRNA in these 10 terms, red represents the upregulated mRNA, and blue represents the downregulated mRNA. **(B)** The primary 10 cellular component (CC) entries that are most significantly enriched. **(C)** The top 10 molecular function (MF) entries that are most significantly enriched.

For the five shared genes, 68 significantly enriched (*P* < 0.05) BPs and 31 CCs as well as 23 enriched MFs were explored by functional enrichment analysis. The top 10 most enriched BP, MF, and CC entries are shown in Figures 2A–C. The significantly enriched KEGG analysis is indicated in Figure 2D.

### Protein–Protein Interaction Network Construction

**FIGURE 3:**
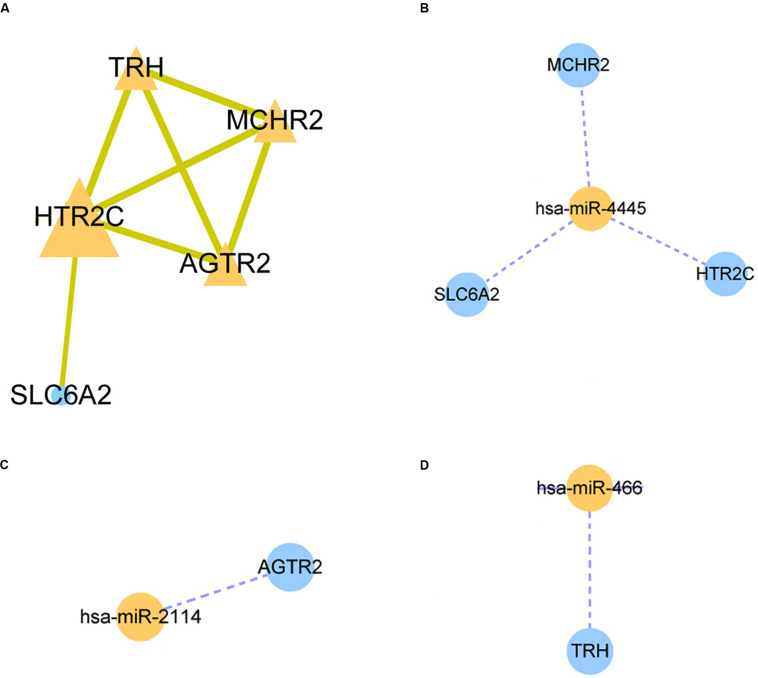
Protein-protein interaction (PPI) network and miRNA-mRNA regulatory network construction. **(A)** The PPI nework for HTR2C. **(B–D)** The miRNA-mRNA regulatory network for hsa-miR-4445, hsa-miR-2114, hsa-miR-466 respectively.

A STRING database was utilized to construct a PPI network for the genes, and the gene interactions with confidence score ≥0.4 were selected for visualization with the Cytoscape software. The MCODE plug-ins were performed to identify significant clustering modules (Figure 3A). The gene with the largest node degree is HTR2C, for which the node degree is 4. The cluster includes HTR2C, TRH, AGTR2, and MCHR2 genes as well as SLC6A2. These four genes except the TRH gene are all downregulated in HCC patients treated with sorafenib only. We believe that these five genes may be key target genes for sorafenib in the treatment of HCC.

### MiRNA–mRNA Regulatory Network

The three candidate miRNAs (hsa-miR-466, hsa-miR-2114, and hsa-miR-4445) were processed to construct a regulatory network and visualized with the Cytoscape software. We found that the three miRNAs interacted with multiple target genes. Among them, hsa-miR-4445 regulates the largest number of target genes (three) (Figure 3B). At the same time, the number of target genes regulated by hsa-miR-2114 is one (AGTR2), the same with hsa-miR-466 (TRH) (Figures 3C,D). Based on these facts, the three selected miRNAs are suggested to be crucial sorafenib-targeted miRNAs in the treatment of HCC.

### The Functional Validation of the Selected Genes

To functionally verify the differences for selected potential genes as well as to explore the possibility of biomarkers for sorafenib treatment in the clinic, we testified concentrations of five target genes in HCC patients’ peripheral blood by using the ELISA method. Compared with those of untreated HCC patients, HTR2C, AGTR2, MCHR2, and SLC6A2 in the sorafenib-treated group display a significantly higher concentration. In contrast, the TRH of the sorafenib-treated group demonstrates a lower expression pattern, which is in line with upstream miRNA expression manner (*P* < 0.05, shown as Table 1).

**TABLE 1:** Selected gene concentrations were compared between two different groups.

	HTR2C (ng/mL, x ± s)	SLC6A2 (ng/mL, x ± s)	MCHR2 (pg/mL, x ± s)	AGTR2 (ng/mL, x ± s)	TRH (ng/mL, x ± s)
untreated	53.17 ± 8.51	47.92 ± 7.36	80.76 ± 14.51	32.86 ± 9.11	41.08 ± 5.36
Sorafenib treated	20.56 ± 4.19	20.46 ± 5.88	60.71 ± 9.52	12.53 ± 4.55	59.66 ± 8.43
*t*	6.15	7.03	1.04	8.34	1.14
*P*	<0.001	<0.001	<0.05	<0.001	<0.05

## Discussion

Hepatocellular carcinoma is the most common primary tumor of the liver, and its mortality is third among all solid tumors, just behind carcinomas of the lung and the colon (Hartke et al., 2017). At this moment, sorafenib seems to be the “jack of all trades” for HCC. However, its overall outcomes are far from satisfactory. The side effects highly hamper its clinical use. In some severe cases, it even causes hypertension, abdominal pain, and discontinuation of therapy (Malik et al., 2019). In addition, due to the genetic heterogeneity of HCC, several patients develop drug resistance to sorafenib (Ahn et al., 2020). All of these are all in dire need of effectively understanding target genes for sorafenib in HCC treatment. To this end, it is beneficial to develop a comprehensive specific expression profile of genes in HCC patients treated with sorafenib only. Here, compared with those of untreated HCC patients, a total of 12 upregulated genes and 54 downregulated genes as well as three DEMs were explored (see Figure 1 for details).

With the use of the PPI network, five primary genes were selected (HTR2C, TRH, AGTR2, MCHR2, and SLC6A2), which are the potential target genes for sorafenib. TRH represents 30–35-nucleotide-long 5′ tRNA-halves (5′ tRHs), which has been reported to play important roles in persistent infections with HBV or HCV. TRH gene abundance correlates with expression of the tRNA-cleaving ribonuclease angiogenin (Selitsky et al., 2015). AGTR2, short for angiotensin II (Ang II) type II receptor, is shown to be overexpressed in HCC tissue using a murine hepatoma model. Suppressing AGTR2 is efficient to inhibit tumor growth time and dose dependently by arresting tumor proliferation, promoting tumor apoptosis, and inhibiting tumor angiogenesis (Lin et al., 2009; Liu et al., 2015). Except for these genes, the others (HTR2C, MCHR2, and SLC6A2) have not been fully explored in HCC, especially in sorafenib-treated patients. So it would be beneficial to further investigate these genes for better evaluating molecular mechanisms as well as the key targets for sorafenib treatment of HCC.

Except for the bioinformatics and machine learning method, we also investigated the selected gene expression from external function study. Clinically, these five genes display a specific expression pattern between two groups, which provide potential biomarkers for sorafenib efficiency in the future. It is worth noting that only TRH shows an increased expression with sorafenib treatment. By the miRNA–mRNA regulatory network analysis here, TRH is the target of hsa-miR-466, which is repressed with sorafenib treatment from differential expression analysis. Sorafenib-treated HCC patients demonstrate upregulated TRH expression level through inhibition of hsa-miR-466. This may be a key molecular mechanism for sorafenib. The same goes for other genes and miRNAs. Next, it will be interesting to study the crosstalk between these miRNAs with the five primary genes in depth.

We further studied the BP related to these genes by using enrichment analysis. We found that these target genes were significantly enriched in BPs related to hormone secretion, ion channel, and cell secretion (see Figure 2 for details). HCC commonly arises from a liver damaged by extensive inflammation and fibrosis. Various factors including cytokines, morphogens, and growth factors are involved in the crosstalk between HCC cells and the stromal microenvironment, which is highly related to ion channel and cell secretion (Villanueva, 2019). Meanwhile, emerging reports suggest a relationship between HCC and thyroid hormone (TH) signaling (dysfunction), raising the possibility that perturbed TH regulation influences the liver microenvironment and HCC formation (Manka et al., 2018), so that these pathways are related to HCC regulated by sorafenib on some levels, and they all deserve further investigation in depth.

To conclude, in the light of increasing dependence on sorafenib for HCC treatment, plus the fact that there remains a limited understanding for sorafenib function, we summarize the differential expression profile of genes and miRNAs in HCC patients treated with sorafenib compared with HCC control patients. Several specific target genes and miRNAs establish specific expression manner, which initiates new insights into the management of sorafenib in clinical use and provides a very valuable data reference for future clinical HCC chemotherapeutic drug research.

## Data Availability Statement

The original contributions presented in the study are included in the article/Supplementary Material, further inquiries can be directed to the corresponding author/s.

## Author Contributions

All authors listed have made a substantial, direct and intellectual contribution to the work, and approved it for publication.

## Conflict of Interest

The authors declare that the research was conducted in the absence of any commercial or financial relationships that could be construed as a potential conflict of interest.

## Footnotes

1 https://tcga-data.nci.nih.gov/tcga/

2 https://string-db.org/

3 https://cytoscape.org/

4 http://mirdb.org/index.html

## Supplementary Material

Supplementary Figure 1 Venn diagram of differentially expressed miRNA target genes and differentially expressed mRNA.
