# Supplementary figures and images for: Exploration of Sorafenib Influences on Gene Expression of Hepatocellular Carcinoma
Source: Front Genet. 2020 Oct 8;11:577000. doi: 10.3389/fgene.2020.577000 (PMC7578401; doi:10.3389/fgene.2020.577000)

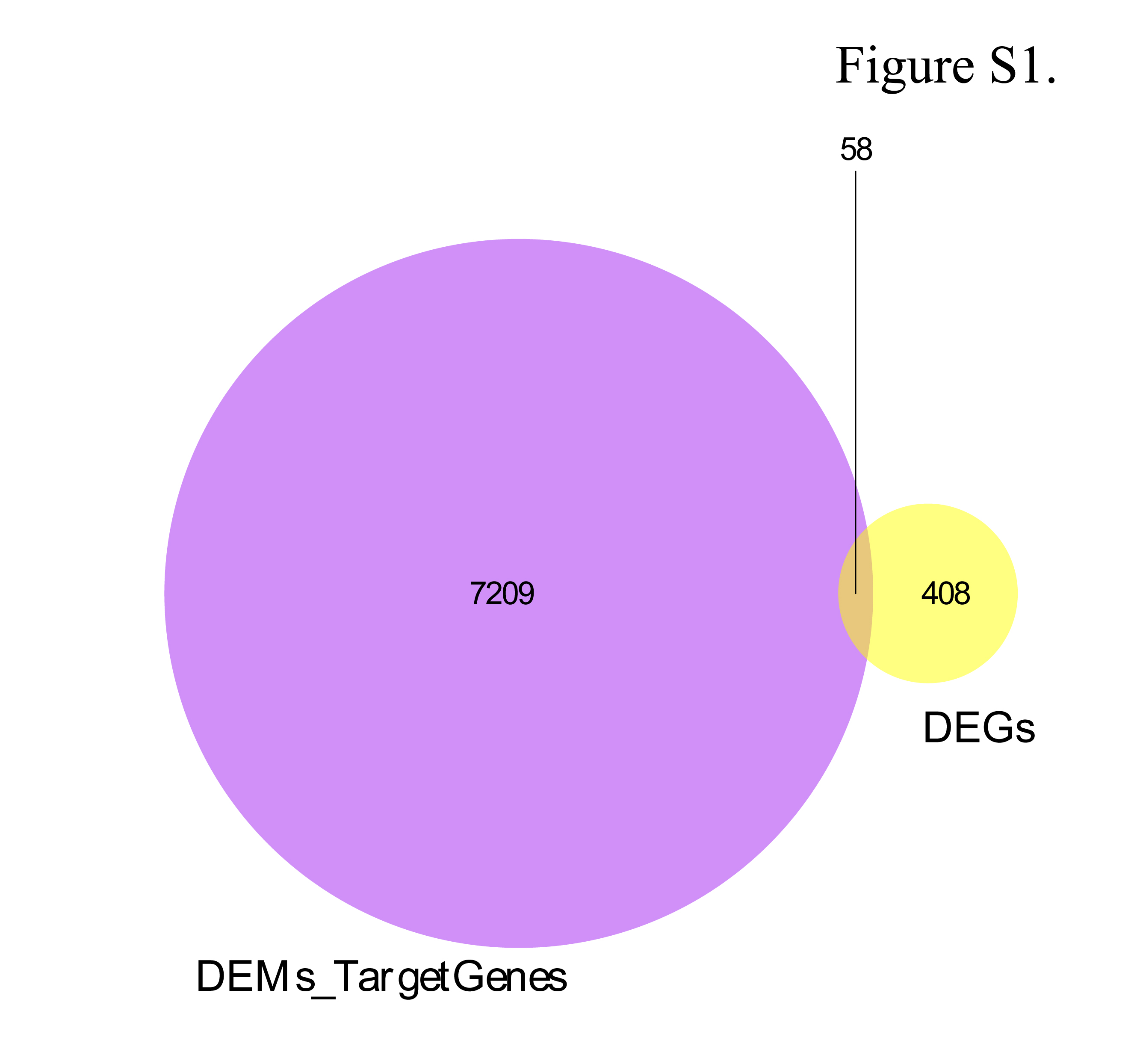

Supplement: Supplementary Figure 1 — Venn diagram of differentially expressed miRNA target genes and differentially expressed mRNA. [file Image_1.jpg]
